# Key disparities between first-generation and continuing-generation medical students: a quantitative analysis

**DOI:** 10.1186/s12909-025-07417-y

**Published:** 2025-07-01

**Authors:** Julian Michael Burwell, Sonia Lobo

**Affiliations:** Department of Medical Education, Geisinger Commonwealth School of Medicine, Geisinger College of Health Sciences, 525 Pine Street, Scranton, PA 18509 USA

**Keywords:** First Generation, Medical School Education, Financial Aid, Basic Needs, Under-Represented in Medicine, Third Party Resources, Visiting Student Learning Opportunities

## Abstract

**Background:**

First-Generation (FG) medical students face significant challenges that can hinder their academic success and well-being. Our study aimed to quantify the disparities between FG and Continuing-Generation (CG) students across four domains including: resilience, social isolation, unmet basic needs, and perception of institutional support.

**Methods:**

An anonymous survey was administered to all four classes of medical students at Geisinger Commonwealth School of Medicine. Students had four weeks to complete the voluntary, 30-question survey which included 29 close-ended questions and one open-ended question to assess challenges in each domain. Survey data were collected in Qualtrics and analyzed using R Epi for R 4.4.1 GUI 1.80 Big Sur Intel build (8416). Primary analysis was performed using Students T-test to evaluate difference in means between cohorts, and one-way ANOVA was used in secondary analysis to correct for confounders between student attitudes across year in school. FG status was defined as having no parent with a 4-year college degree. Seventy-two students responded, and 62 completed surveys were included in the analysis.

**Results:**

Among the 62 respondents (15 FG, 47 CG), FG status predicted increased resilience (*p* = 0.01), feelings of social isolation (*p* = 0.005), and unmet basic needs (*p* < 0.001). Key disparities included food/housing insecurity, affordability of educational resources, and access to visiting student learning opportunities. There were no significant differences in resilience (*p* = 0.656), social isolation (*p* = 0.656), basic needs (*p* = 0.07), or perception of institutional support, (*p* = 0.651) based on year of training. The findings highlight FG students’ financial strain, disconnection from peers, and desire for targeted support. Institutional scholarships received by 47% of FG and 53% of CG students (χ2 = 8.4e-31, *p* = 1.0) mitigated but did not eliminate these disparities.

**Conclusion:**

Our data reveal substantial, ongoing challenges faced by FG medical students and support the previous findings identified by Havemann et al. We suggest that by enhancing healthcare access, grants for educational expenses, subsidized study materials, and fostering peer networks, medical schools can proactively address these gaps in medical student success to build a physician workforce equipped to serve all patients and communities.

**Supplementary Information:**

The online version contains supplementary material available at 10.1186/s12909-025-07417-y.

## Background

First Generation (FG) students (defined as those whose parents did not graduate with a four-year degree) face many challenges. Research has shown that FG students apply to Doctor of Medicine (MD) and Doctor of Osteopathic Medicine (DO) medical schools at a lower rate, are accepted at a lower rate and further, of those accepted, fewer matriculate compared to their Continuing-Generation (CG) peers [1–3]. Once in medical school, FG students report significantly lower scores on measures of well-being including self-care, environmental quality of life, perceptions of medical school support for family and personal responsibilities, and sleep quality [4, 5]. American Association of Medical Colleges (AAMC) data have also demonstrated that FG students report higher levels of stress, fatigue, and financial concerns than their CG peers [6].

Based on these concerns Havemann and colleagues interviewed FG students from 27 medical schools across the United States (US) [7]. Their qualitative analysis identified four themes common among FG students that made medical school especially challenging: (1) isolation and exclusion related to being newcomers to medicine; (2) difficulty accessing basic resources (e.g., food, rent, transportation) and educational resources; (3) a lack of faculty or institutional support to address these challenges; and (4) a reliance on grit and resilience to survive [7]. *Grit* has been defined as “perseverance of effort” despite setbacks. However, studies of 4-year undergraduate students have demonstrated that grit does not necessarily correlate with academic success, while institutional support does [7, 8]. It has been suggested that this is in part due to the exogenous financial burden that FG students bear. Social isolation has been shown to negatively affect students’ negative health based on an experience of social class difference between themselves and the perceived norms of an institution [7, 9]. Many FG students report unmet basic needs, including food and rent, as well as required educational expenses such as board examinations and study materials. Some FG students have even reported working additional jobs, like substitute teaching or selling plasma, to make ends meet [7, 10]. The perception of a lack of support in the “Ivory Tower” is an ongoing phenomenon that FG students face. FG students report that these struggles last beyond their education, with their peer group shrinking as they advance in medical training [11]. FG students have been shown to be more averse to taking on student loans and report higher levels of debt upon graduating [8, 12]. In the last 20 years, the average cost per semester of an MD program in the US has increased from $18,048 to $29,484, which places FG students at a unique disadvantage [13–15]. To address these issues, the AAMC has advocated for greater visibility of FG in medicine, but enrollment rates among FG students remain low [3, 16, 17].

Building upon Havemann’s framework, we sought to quantitatively assess how the four themes impacted FG medical students, with particular emphasis on the specific financial need differences between groups. As an institution committed to supporting FG students, Geisinger Commonwealth School of Medicine (GCSOM) presented an ideal setting to apply quantitative analysis. Based on our survey of the literature, we hypothesized that FG students at GCSOM would report greater levels of concern in these domains compared to their CG peers, independent of their stage of training or receipt of institutional scholarships.

## Methods

### Study design

**Fig. 1:**
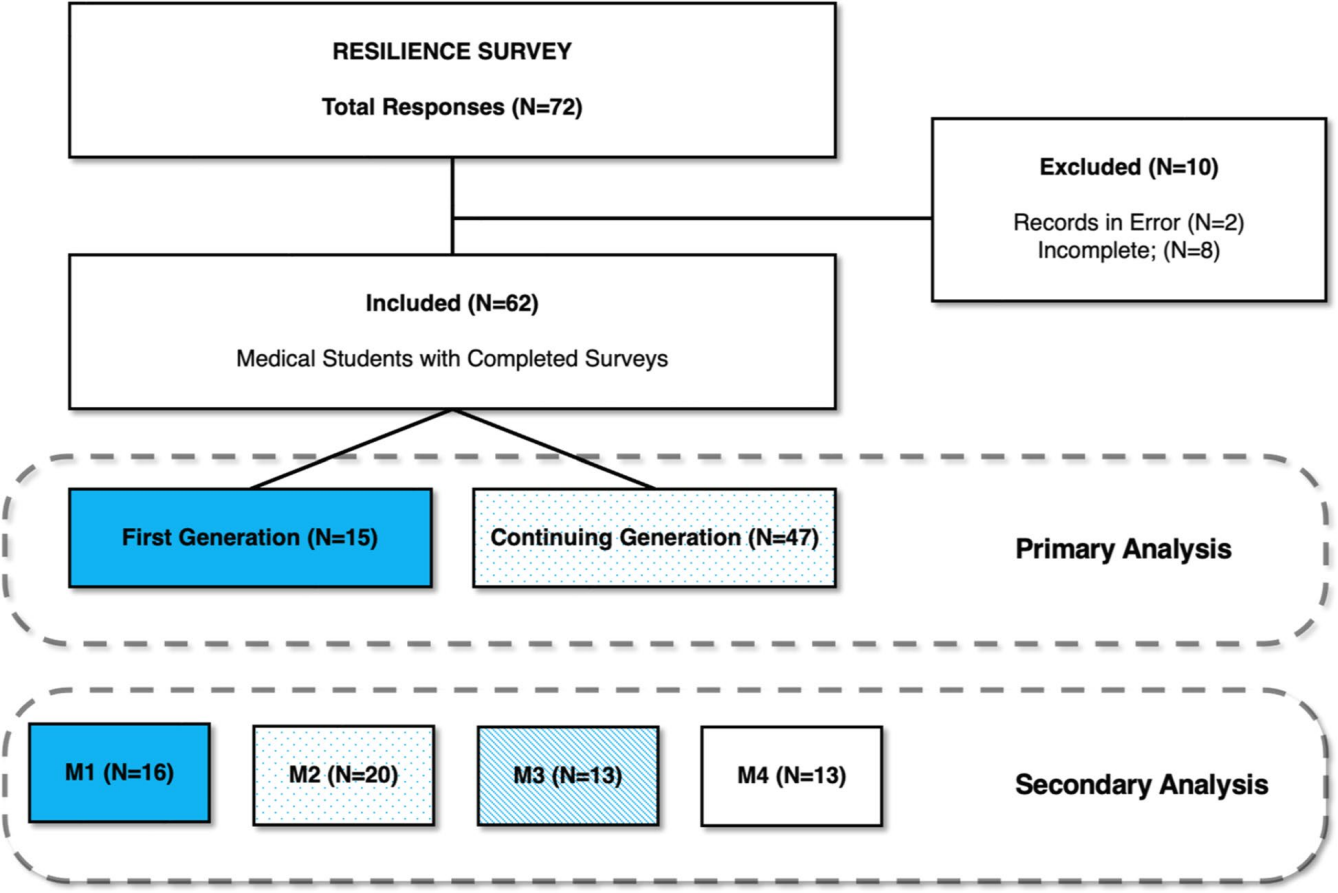
Study Design

To test this hypothesis, we anonymously surveyed current GCSOM medical students using close-ended questions derived from the four key themes identified by Havemann et al*.* and compared responses between the FG and CG cohorts. We created an anonymous, 30-item survey (see Additional file 1). FG status was defined by the AAMC definition as having parents who did not graduate with a four-year college degree, while CG students had at least one parent with a four-year degree. Low-income (LI) status was defined by the AAMC definition of growing up in a household where the total family annual income was equal to or less than 400% of the national poverty level per family size [18]. Close-ended questions were intended to measure the extent to which the four themes identified by Havemann et al. including, reliance on grit (resilience), feelings of social isolation, unmet basic needs, and perception of institutional support, impacted FG medical students [7]. Additional questions included basic demographic information, identified FG vs CG status based on parental education, and one open-ended question about the personal meaning of becoming a doctor to ensure students of all backgrounds had the opportunity to describe more details about their journey [19]. The survey was developed using Qualtrics software and tested by the research team to ensure continuity [20]. Improvements were made through iterative discussions. The survey was administered to all four classes of GCSOM medical students (456 students: 114 MD1 s; 112 MD2 s; 114 MD3 s; 116 MD4 s) over a 4-week period from December 2024 to January 2025. Potential participants were invited to complete the online survey via email, with one follow-up email sent as a reminder. Participation was voluntary, and notably, students were blinded to the study objective of comparing responses between the FG and CG groups (Fig. 1). The study was approved and deemed exempt by the Geisinger Institutional Review Board, 2024–0482**.**

Data reporting included tables which compared survey response differences between FG and CG groups. Weighted survey responses were averaged across groups in the four domains for visualization and compared statistically. Further examination of key differences included cohort differences in Social Benefits by Student Background, Personal Support Structures of FG and CG Medical Students, Anticipated Debt upon Graduating by Medical Student Background, Financial Savings by Medical Student Background, and finally select responses to the open question, “What will it mean to you when I can say, ‘I am a physician’?”.

### Data analysis

Items were assessed using a 5-point Likert scale (1 = “not a concern” to 5 = “strong concern”) [21]. Questions were grouped into four thematic categories: resilience (4 items, score range 4–20), isolation (6 items, score range 6–30), basic needs (7 items, score range 7–35), and institutional support (5 items, score range 5–25). For each participant, items within each category were summed to create raw category scores. All statistical analyses were performed on raw scores, with results subsequently normalized to a 0–100 scale for reporting purposes using the formula: normalized score = ((raw score—minimum possible score)/(maximum possible score—minimum possible score)) × 100. On the normalized scale, 50 represents the neutral midpoint (corresponding to an average item score of “3” on the original Likert items). Scores above 50 indicate increasing levels of concern, while scores below 50 indicate decreasing levels of concern. Internal validity of survey responses was checked utilizing Cronbach’s alpha with additional assessment performed with signal to noise ratios.

For primary analysis, normality of the summed scores were assessed with QQ plots and Shapiro–Wilk tests [22]. After confirming normal distribution, cohorts were compared using Student’s t-test. Alternative analyses were performed to control for potential confounding sources including potential errors in student bias due to the Abigail Geisinger Scholar’s loan-forgiveness program, which allows students to have a subsidized four-year education on a limited stipend in exchange for equal years’ service payment upon completion of residency [23, 24]. Chi-squared tests were used to compare categorical variables between groups. Fisher’s Exact test was used to correct for *p*-values where there were less than five occurrences in each series [25].

For secondary analysis, one-way ANOVA was used to test if themes differed by year in school (M1-M4) [26]. It is well established that students have different perceptions and needs as they progress in medical education, largely due to the transition from the classroom to the clinical space. We intended to isolate the effects of students’ FG status from their status as an M1 versus M2 versus M3 versus M4 [27]. Epi for R 4.4.1 GUI 1.80 Big Sur Intel build (8416) was used for statistical analysis [28]. *P*-values for statistical significance were set at < 0.05.

## Results

The survey was emailed to 456 medical students and 72 responded to the survey; 62 completed surveys were included in the analysis. The results had incomplete responses, and given the limited sample size, imputation was deemed a non-viable strategy in this cohort. Most respondents identified their educational background as CG (77%) with balanced participation across classes (MD1 = 26%; MD2 = 32%; MD3 = 21%; MD4 = 21%) (Table 1). The effect of the Abigail Scholar’s Program was non-contributory in both cohorts, FG Abigail Scholars (*n* = 7, 47%) and CG Abigail Scholars (*n* = 24, 51%) (χ^2^ = 8.4166e-31, *p* = 1.0). Survey responses demonstrated internal validity (Cronbach’s α = 0.79, 95% CI 0.72–0.86), and a signal-to-noise Ratio (S/N = 3.8).

**Table 1:**
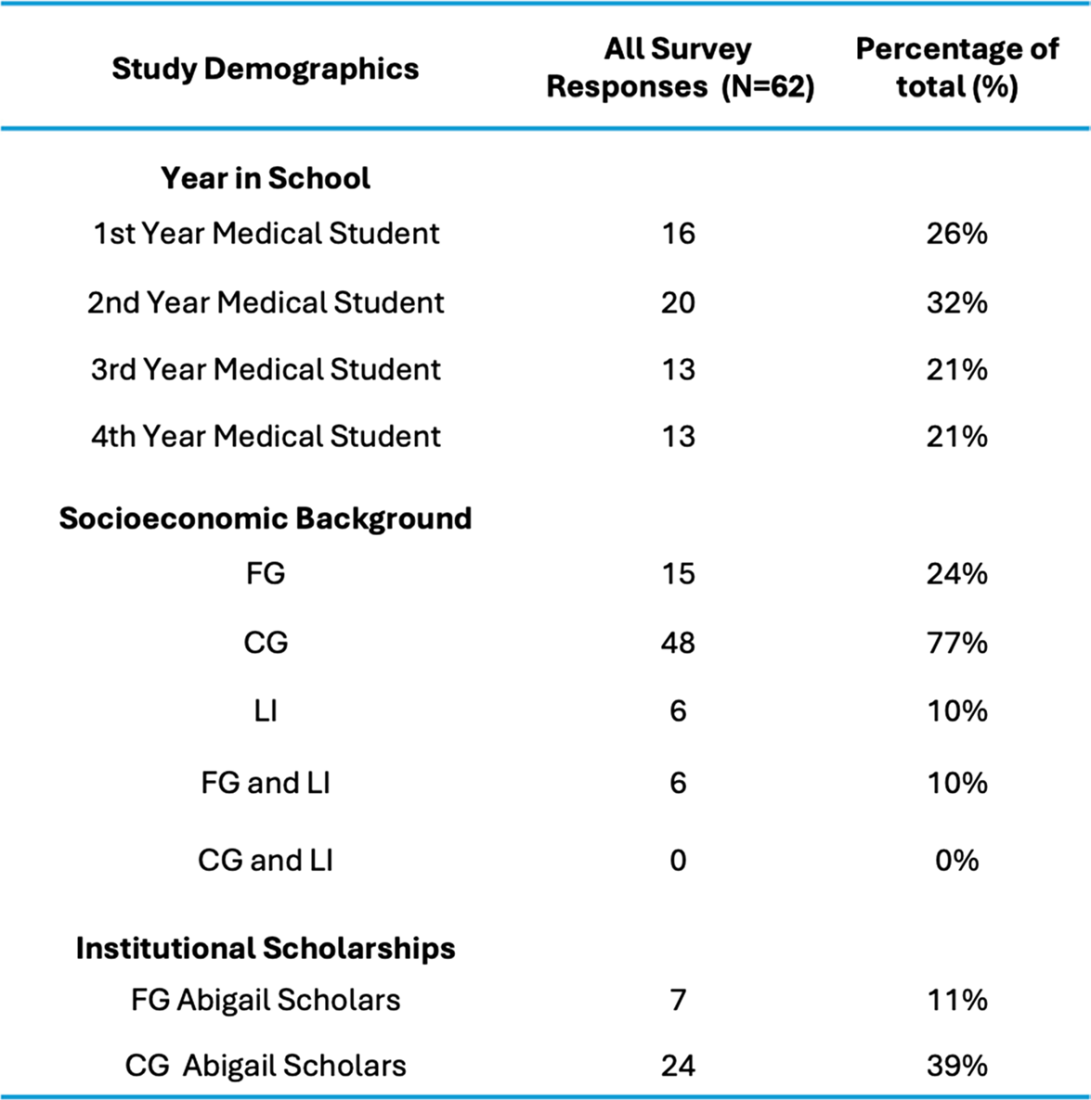
Study demographics

### Primary analysis of student survey responses by FG vs. CG status

**Fig. 2:**
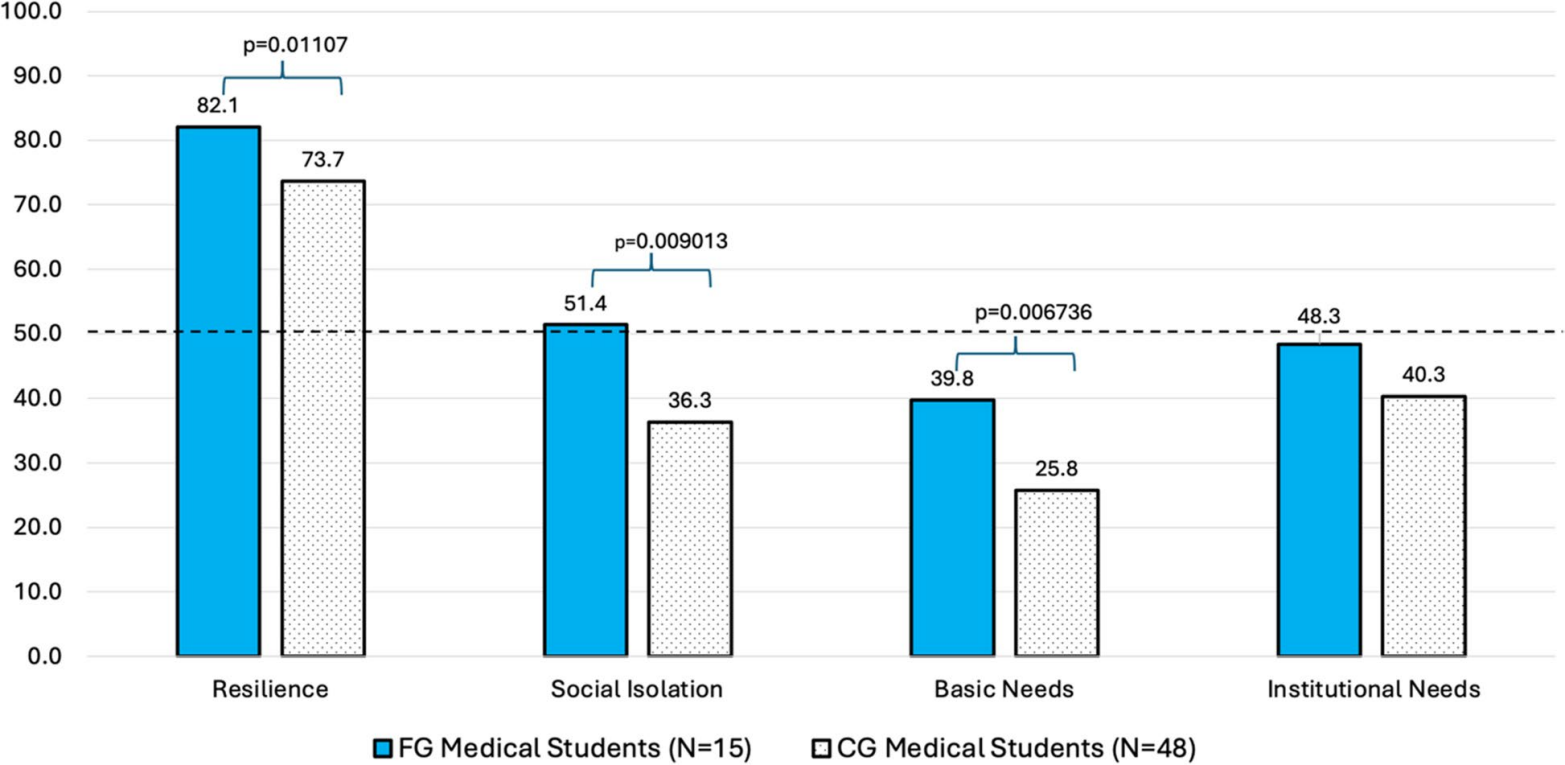
Themes by Medical Student Background (averages shown)

Our analysis demonstrated strong resilience in both cohorts, with FG students reporting greater levels of resilience (t = 2.6204, *p* = 0.01107), feelings of social isolation (t = 2.6978, *p* = 0.009013), and difference in unmet basic needs (CG, t = 2.8053 *p* = 0.006736) (Fig. 2). Institutional needs were not a major area of concern for either group and not statistically significant between FG and CG students (t = 1.6436, *p* = 0.1054). FG students cited many more gaps in basic needs support compared to their CG peers (Table 2). Personal support networks were similar between cohorts (Table 3). The anticipated debt upon graduation was similar between cohorts; however, CG students reported higher amounts of debt (> $400,000), but these results were not statistically significant (*p* = 0.1552) (Table 4).

**Table 2:**
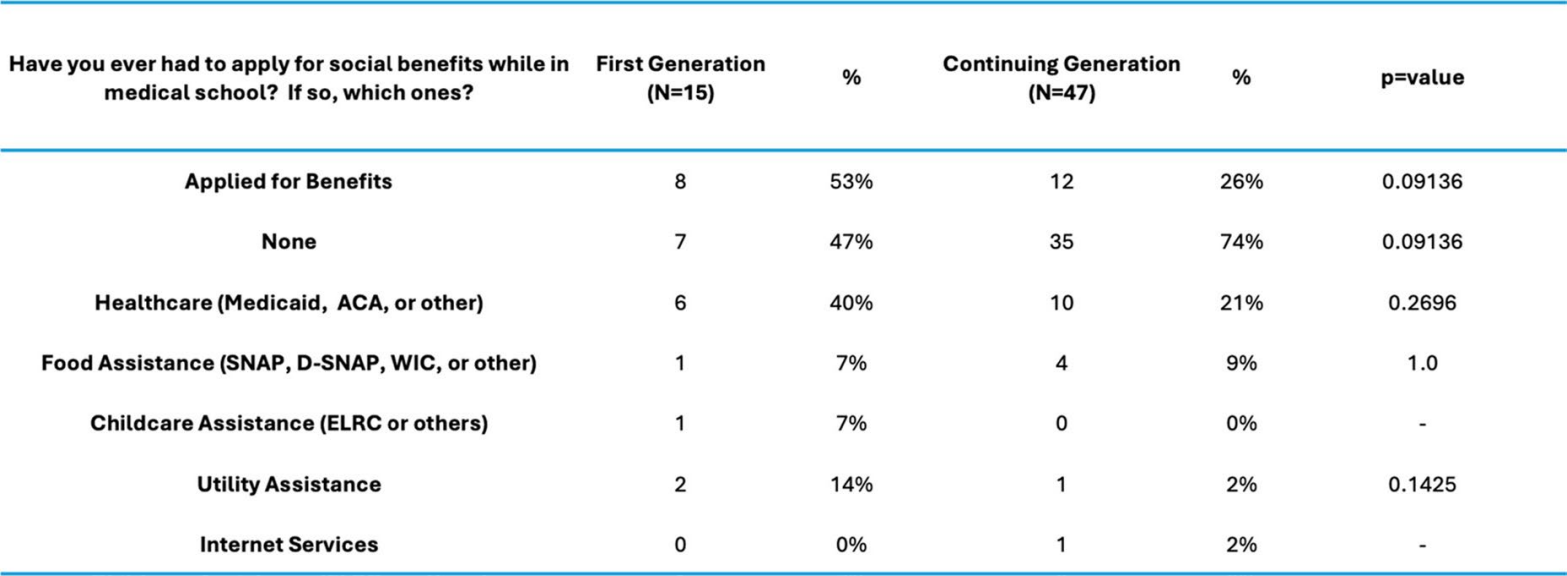
Social benefits by student background

**Table 3:**
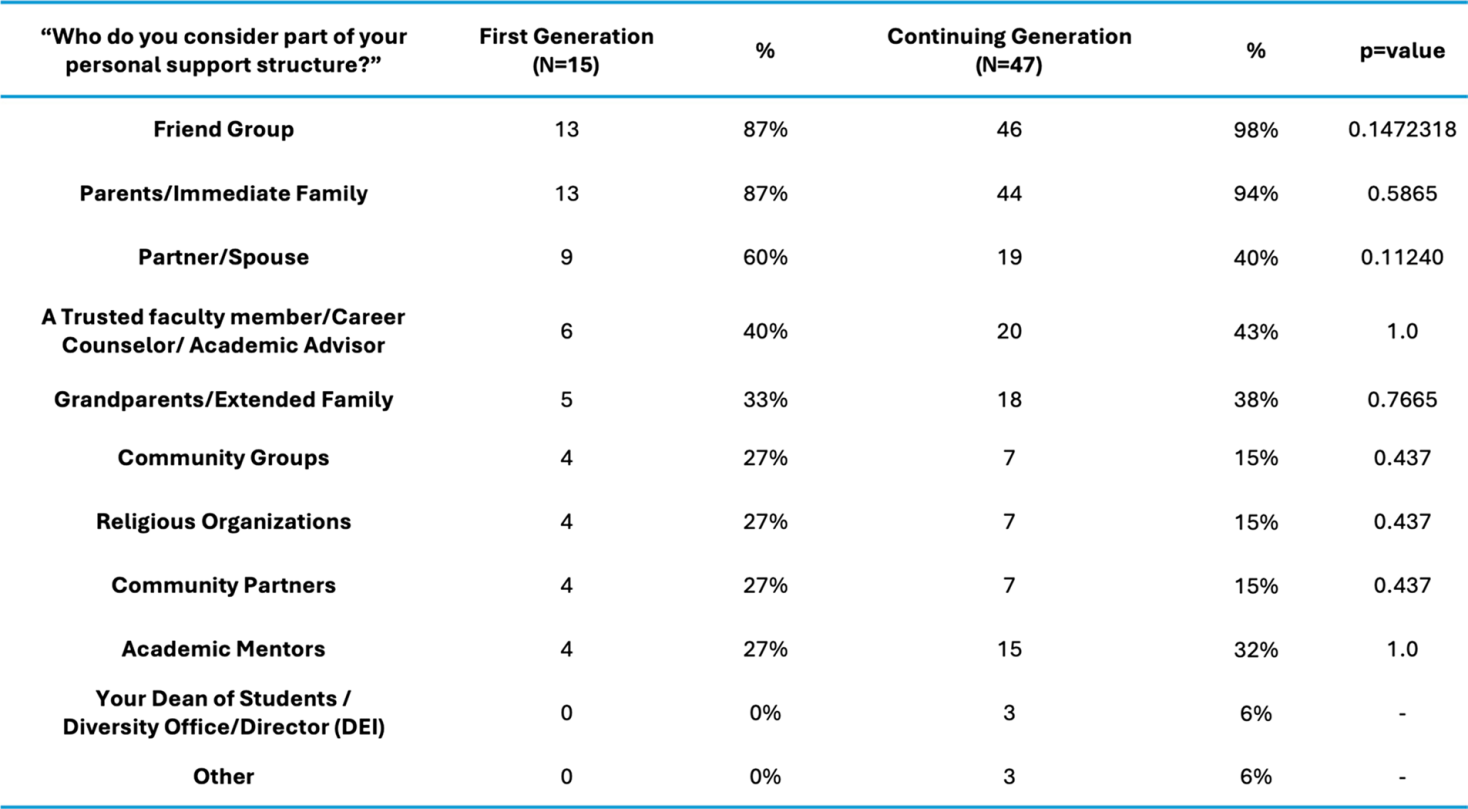
Personal support structures of FG and CG medical students

**Table 4:**
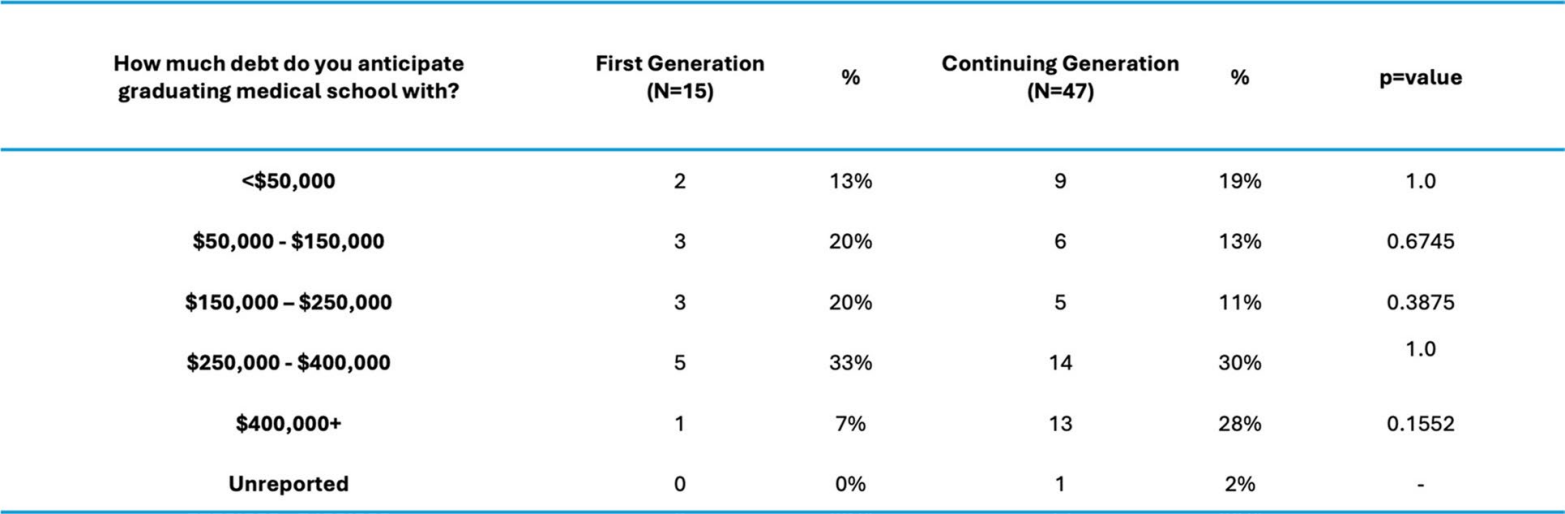
Anticipated debt upon graduating by medical student background

### Measures of financial disparity

After primary analysis, we sought to capture more specific views on personal experience of financial disparity between our two cohorts by focusing on specific student needs. In the following questions we highlight several significant differences. First, when asked “*If your financial aid were interrupted, how long would you be able to pay for your basic needs such as housing, food, healthcare, and transportation? (excluding educational expenses),”* 40% of FG students reported that they would only be able to survive 1–2 weeks (FG 6/15 (40%) vs CG 5/47 (11%), χ2 = 4.8558, *p* = 0.01758) (Table 5). Second, when asked “*How often do financial constraints impact your ability to purchase third-party resources for medical education?”* the results were significant (FG 3.8 vs CG 2.937, t = 2.527, *p*-value = 0.01411). Lastly, when asked “*If you are considering a VSLO (Visiting Student Learning Opportunity), how confident are you that you could pay for it*?”, we observed a major divergence of opinions with FG students landing in the “Unconfident Range” and their CG peers expressing moderate to confident range in their ability to pursue VSLOs (FG 3.733333 vs. CG 2.723404, t = 2.9431, *p*-value = 0.004615).

**Table 5:**
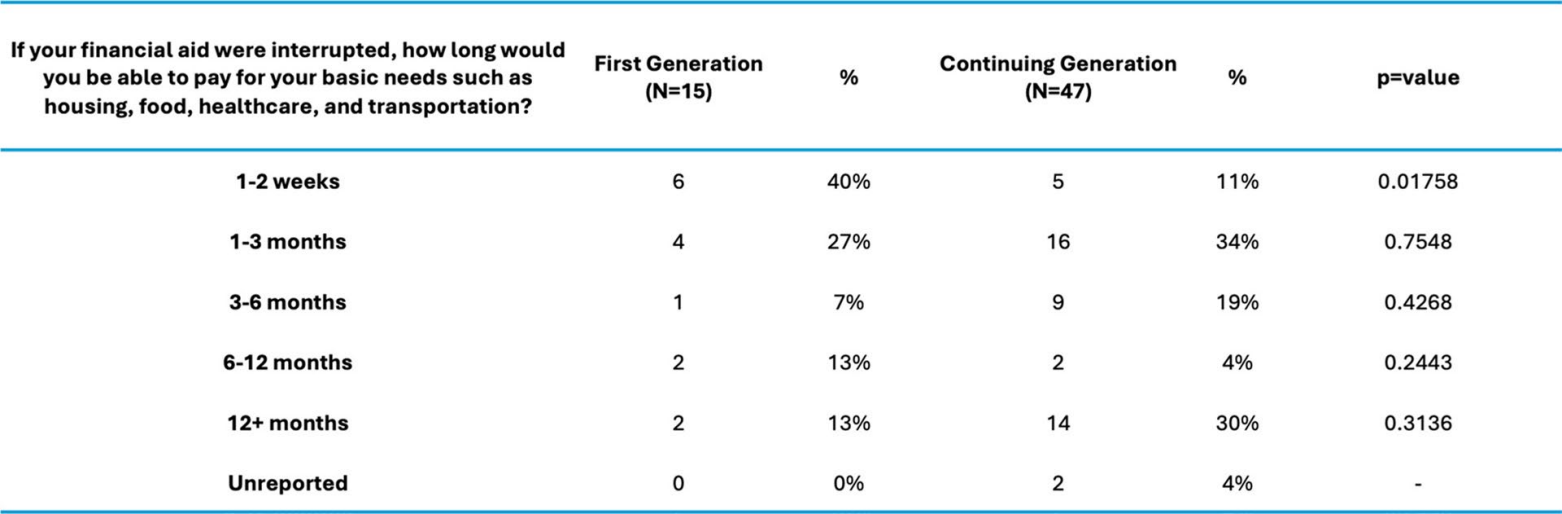
Financial savings by medical student background

### Secondary analysis of student survey responses by year in medical school

**Fig. 3:**
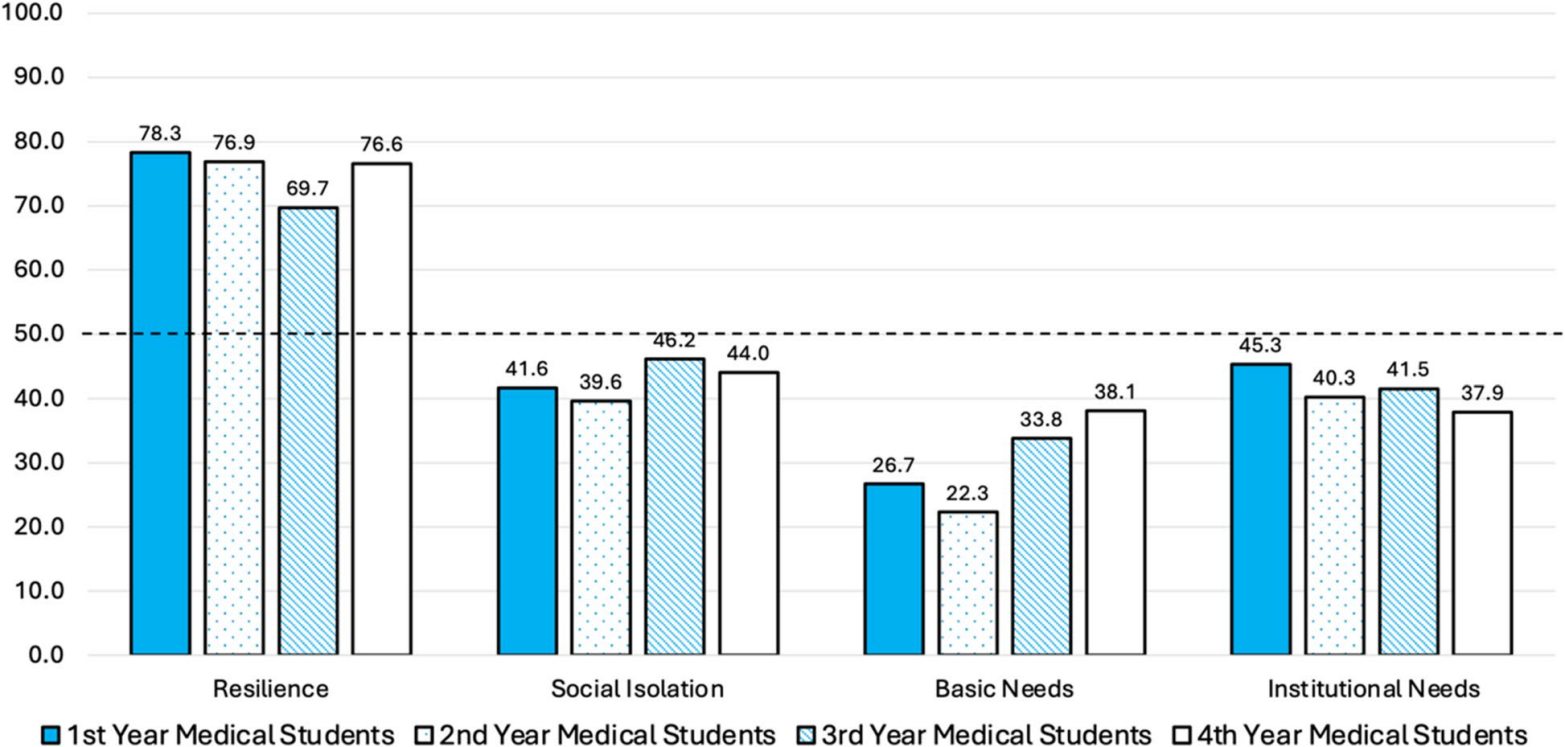
Secondary analysis of characteristics by year in medical school (averages shown)

Students were reassorted and one-way ANOVA tests were conducted to compare the scores of students across each of the four key themes across the four years of medical school training (M1-M4). Students reported similar levels of resilience (F(3, 59) = 0.541, *p* = 0.656). The extent of social isolation experienced did not vary significantly across the M1 to M4 years (F(3, 59) = 0.541, *p* = 0.656). Medical students reporting increasing levels of unmet basic needs correlated with their level of training, but the results were not statistically significant (F(3, 59) = 2.479, *p* = 0.07). Finally, our analysis found no significant differences in perception of institutional support based on year in medical school (F(3, 59) = 0.549, *p* = 0.651). Based on our secondary analysis to adjust for confounding variables, measures of resilience, social isolation, basic needs, and institutional needs remained consistent across the four years of medical education. We identified these as non-confounding for the purposes of our survey analysis comparing FG students to their CG peers (Fig. 3).

## Discussion

Our quantitative assessment of the FG student experience supports the qualitative analysis by Havermann et al., revealing substantial ongoing challenges across themes of resilience, social isolation, and unmet basic needs. These differences are further highlighted by some of the qualitative data captured by our survey (Table 6). Notably, FG students did not report significant differences in their perception of institutional support. We believe this may be attributed to effective strategies implemented by our institution such as FG social events, an FG committee, an FG Chair for each MD class who sits on Student Council, and an annual FG Day of celebration which collectively help to support FG students [29, 30]. Our data suggests nonsignificant differences in overall anticipated debt but significance in more day-to-day needs experienced by FG students, including study resources and basic needs.

**Table 6:**
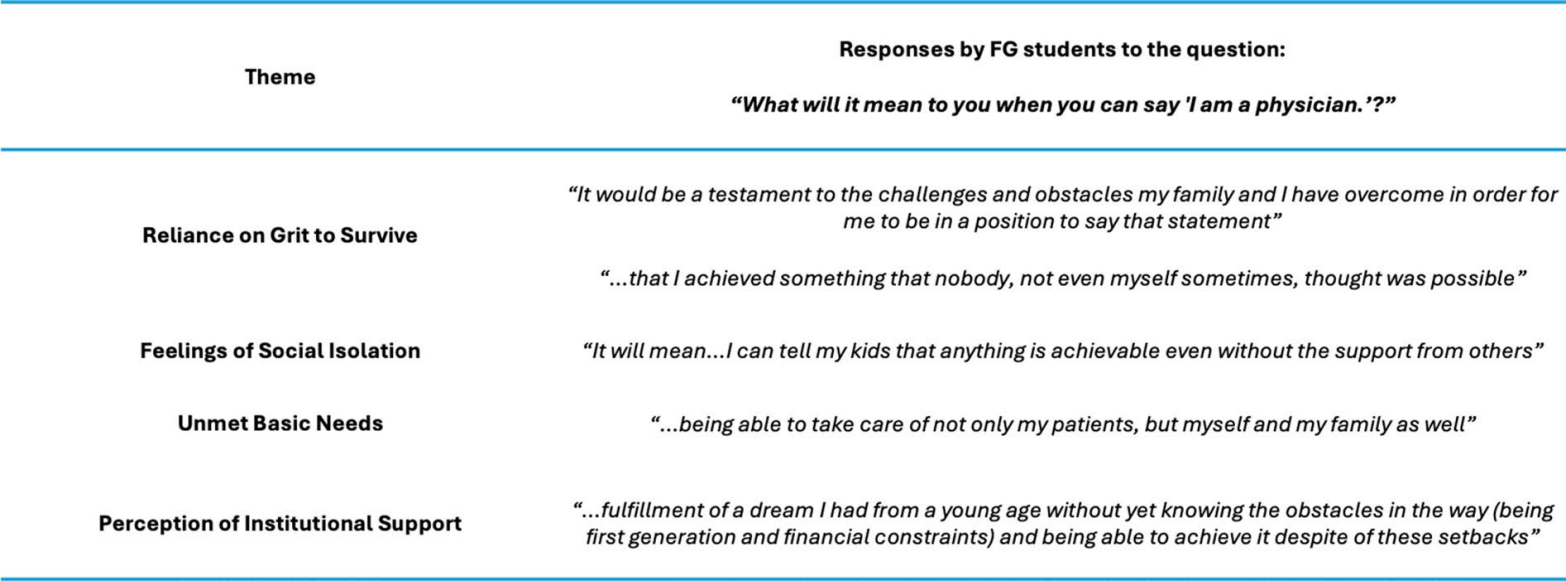
Select responses first generation students

Our findings suggest that more targeted intervention strategies such as basic needs assistance, VSLO scholarships, and third-party study resource assistance may be more meaningful ways to help FG medical students than addressing the overall debt ceiling. The lack of basic needs support was significant (*p* = < 0.01) in our study. It should be of concern to medical professionals that there are students who are unable to progress through medical school without relying on federal or state funding support. This was true even in our CG cohort, of whom twenty percent of students needed federal and state aid to support their healthcare. Despite taking out loans or receiving financial assistance, there were still unmet needs reported by these students, including housing insecurity, food insecurity, utilities and security, and difficulty with childcare (Table 2).

The basic needs stability of FG students is an area where relevant stakeholders could have a unique opportunity to invest in the future of medicine. There are other opportunities as well: for instance, VSLO offers students the chance to gain important clinical experiences and grant access to prestigious residency programs [31]. Access to third-party resources, such as study materials and educational tools, has also become ubiquitous in medical school and can influence academic performance. These resources can be expensive, ranging anywhere from $100-$479 for a 12-month subscription [32, 33]. FG students have been shown to demonstrate greater risk aversion, potentially making them reluctant to take on additional student loans even when available. We believe this cautious approach to risk and reward in borrowing decisions may impact FG students more than their peers.

The American Medical Association (AMA) [34] and AAMC have called for increased visibility for FG students by using metrics like Integrated Holistic Student Affairs (IHSA) model to extend the principles of holistic review during the admissions process to encompass all aspects of a student’s experience on campus, addressing their individual needs and circumstances [35]. However, there appears to be a lack of ideas on how to implement meaningful change for students across multiple years medical school where the intrinsic challenges of medical education can be difficult enough. Our findings encourage more tailored options for US medical schools seeking to improve the attractiveness of their program for FG applicants.

Our study has several strengths including a quantitative approach that was able to identify specific, actionable interventions to support FG students. Additionally, by blinding participants to the study’s focus on FG status, we likely reduced reporting biases and obtained more candid responses. By framing the study around resilience, participants were not primed to frame their experiences in terms of FG vs. CG. Our findings align with those from other studies on the FG student population, suggesting the themes identified by Havemann and colleagues provide a reliable framework for understanding the FG experience that transcends any single institution; moreover, we believe our findings build on this framework highlighting the magnitude of the day-to-day challenges faced by FG students and identifying specific opportunities for medical schools [36].

Our survey is limited by possible self-reporting bias, a limited sample size, and a substantial number of students in the Abigail Geisinger Scholars Program. Efforts were made to minimize bias by ensuring anonymity, testing internal validity, and performing secondary analyses to demonstrate that these institutional biases could be corrected for and explained. Furthermore, our study provides initial quantitative evidence that FG student support initiatives may be warranted, due to the ongoing unmet needs experienced by both FG and CG students across all years of training.

Several questions remain from this study that future research could be aimed toward: first, more direct measures connecting FG status to specialty placement (primary care vs specialization); and second, a quantitative analysis to see if bridging gaps in basic needs improves academic performance of FG students. By addressing these topics, medical training could become more egalitarian, focusing on an individual’s personal strengths rather than intergenerational wealth [37, 38].

## Conclusion

Our study reveals challenges faced by FG medical students, corroborating the qualitative analysis by Havemann et al. across themes of unmet basic needs, feelings of social isolation and exclusion, and reliance on grit to survive. Importantly, these findings were replicated quantitatively in a small sample size and at an institution with very different generational profiles than those of the original study. FG interventions at our institution such as FG social groups, FG special events, FG representation on Student Council, and school-wide FG recognition could account for the non-difference we observed in student perceptions of institutional needs. However, despite these interventions, our data revealed differences in students’ financial needs, including lack of healthcare coverage, limited access to third-party resources, and funding for visiting student learning opportunities. We believe that FG students at other US medical schools could benefit from targeted interventions such as: 1) basic needs support including school subsidized medical care plans for FG students and their families; 2) VSLO scholarships for FG students pursuing out of state medical training; 3) third-party resource support including FG specific stipends for study recourses like UWorld, AMBOSS, and USMLE practice exams; and 4) FG social events to help reduce loneliness and facilitate unity across classes. Future research should evaluate the effectiveness of these support strategies, aiming to develop a physician workforce equipped to serve all patients and communities.

## Supplementary Information

Supplementary Material 1.

## Data Availability

Data is available upon request from the corresponding author.

## Abbreviations

AAMC: American Association of Medical Colleges
AMA: American Medical Association
CG: Continuing generation
DO: Doctor of Osteopathic Medicine
FG: First generation
GCSOM: Geisinger Commonwealth School of Medicine
IHSA: Integrated Holistic Student Affairs
LI: Low income
MD: Doctor of Medicine
US: United States
USMLE: United States Medical Licensing Examination
VSLO: Visiting student learning opportunity
