# Supplementary material for: Key disparities between first-generation and continuing-generation medical students: a quantitative analysis
Source: BMC Med Educ. 2025 Jul 1;25:955. doi: 10.1186/s12909-025-07417-y (PMC12210446; doi:10.1186/s12909-025-07417-y)
Supplement: Supplementary file 1 — Supplementary Material 1. [file 12909_2025_7417_MOESM1_ESM.pdf]

# Resilience in Medical School

Start of Block: Introduction

Introduction Resilience in Medical School   We are interested in understanding the experiences of medical students. As a valued member of our medical learner community, we request your assistance by completing this voluntary survey which has the potential to help medical students just like you at a national level. This survey is not mandatory, and you can withdraw at any time by simply closing the browser window. Your responses will be kept strictly confidential and only used for this study. No identifying information will be collected about you. This is a 26 Question Survey and should take approximately 10 minutes to complete. If you have any questions or concerns, please contact Julian Michael Burwell (jburwell1@som.geisinger.edu). By clicking NEXT PAGE you are verifying that you have read the explanation of the study, and that you agree to participate.   Thank you for your time and consideration.

End of Block: Introduction

---

Start of Block: Reliance on Resilience

Section 1 Reliance on Resilience:   First, we would like to learn more about your personal academic experience.

---

Q1.1 How often do you find yourself adapting to unexpected situations without external assistance?

- ○ Never  (1)
- ○ Sometimes  (2)
- ○ About half the time  (3)
- ○ Most of the time  (4)
- ○ Always  (5)

---

Q1.2 How confident are you in your ability to bounce back from setbacks?

- Not at all confident (1)
- Unconfident (2)
- Somewhat confident (3)
- Confident (4)
- Extremely Confident (5)

---

Q1.3 How strongly do you agree with the following statement: "My academic success is a result of my effort"?

- Strongly Disagree (1)
- Disagree (2)
- Neither Yes or No (3)
- Agree (4)
- Strongly Agree (5)

---

Q1.4 How often do you seek emotional support from peers or mentors when facing challenges?

- Never (1)
- Sometimes (2)
- About half the time (3)
- Most of the time (4)
- Always (5)

---

Q1.5 To what extent do you believe your resilience contributes to your ability to overcome obstacles?

- Not at all (1)
- Slightly (2)
- Moderately (3)
- Quite a bit (4)
- Extremely (5)

End of Block: Reliance on Resilience

---

Start of Block: Isolation and Exclusion

Introduction Isolation and Exclusion: The next few questions are about your personal and academic experience in medical school.

---

Q2.1 How often do you feel a sense of belonging at your medical school?

- Never (1)
- Rarely (2)
- Sometimes (3)
- Often (4)
- Always (5)

---

Q2.2 Conversely, how often do you feel like an outsider in medical school?

- Never (1)
- Rarely (2)
- Sometimes (3)
- Often (4)
- Always (5)

---

Q2.3 How often do you feel left out or excluded during group activities or discussions?

- Never (1)
- Rarely (2)
- Sometimes (3)
- Often (4)
- Always (5)

---

Q2.4 How often to you feel excluded from social activities or events?

- Never (1)
- Rarely (2)
- Sometimes (3)
- Often (4)
- Always (5)

---

Q2.5 How often do you feel you lack access to mentors who understand your background?

- Never (1)
- Rarely (2)
- Sometimes (3)
- Often (4)
- Always (5)

**End of Block: Isolation and Exclusion**

---

**Start of Block: Access to Resources (Basic and Educational):**

Section 3 Access to Resources (Basic and Educational): The questions in this section are designed to gain your thoughts about access to resources in your day-to-day life as a medical student.

Q3.1 How easy is it for you to access additional study materials beyond the official medical school curriculum?

- Very easy (1)
- Easy (2)
- Neither easy nor difficult (3)
- Difficult (4)
- Very Difficult (5)

Q3.2 How often do financial constraints impact your ability to purchase third-party resources for medical education?

- ○ Never (1)
- ○ Rarely (2)
- ○ Sometimes (3)
- ○ Often (4)
- ○ Always (5)

---

Q3.3 Have you ever had to apply for social benefits (such as Federal, State or Local assistance in the form of Medicare/Medicaid, Housing Assistant, Food Assistance (SNAP), Childcare assistance (ELRC), Help with Utility Bills, or Temporary Assistance for Needy Families (TNAF) while in Medical School?

- ○ Yes (1)
- ○ No (2)

*Skip To: Q3.4 If Have you ever had to apply for social benefits (such as Federal, State or Local assistance in the... = No*

---

Q3.3.1 If yes to the previous question, what social programs have you used (select all that apply)?

▢ Medicare (1)

▢ Medicaid (ACA, State subsidized Healthcare) (2)

▢ Food Assistance (SNAP, D-SNAP, WIC, etc.) (3)

▢ Childcare Assistance (ELRC, others) (4)

▢ Housing Help (rental and buyer assistance program) (5)

▢ Utility Assistance (local or state programs to subsidize healthcare) (6)

▢ Other (please specify) (7) ________________________________________________

---

Q3.3.2 If yes to the previous question, how often have you had to utilize social programs to get through school? (e.g., Medicare/Medicaid, SNAP, ELRC, etc.)

○ Not Applicable (1)

○ Once (2)

○ A few times (3)

○ Most of my education (4)

○ During all of my education (5)

---

Q3.4 If your financial aid were interrupted, how long would you be able to pay for your basic needs such as housing, food, healthcare, and transportation? (Not including educational expenses)

- ○ 1-2 weeks (1)
- ○ 1-3 months (2)
- ○ 3-6 months (3)
- ○ 6-12 months (4)
- ○ 12+ months (5)

---

Q3.5 How frequently do you skip meals due to lack of funds?

- ○ Never (1)
- ○ Rarely (2)
- ○ Sometimes (3)
- ○ Often (4)
- ○ Always (5)

---

Q3.6 How often does food insecurity affect your academic performance?

- ○ Never (1)
- ○ Rarely (2)
- ○ Sometimes (3)
- ○ Often (4)
- ○ Always (5)

End of Block: Access to Resources (Basic and Educational):

Start of Block: Institutional Support

Group 3 Institutional Support: Next, we would like to ask some questions about your experience and support structure in medical school.

Q3.1 To what extent do you feel your school is invested in your personal success?

- ○ Not at all (1)
- ○ Slightly (2)
- ○ Moderately (3)
- ○ Quite a bit (4)
- ○ To a Great Extent (5)

Q3.2 Who do you consider part of your personal support structure? SELECT ALL THAT APPLY:

- ▢ Friend Group (1)
- ▢ Partner/Spouse (2)
- ▢ Parents/Immediate Family (3)
- ▢ Grandparents/Extended Family (4)
- ▢ Community Groups (Sports group, Religious Organizations, Community Partners, etc) (5)
- ▢ Academic Mentor(s) (Professor or PI) (6)
- ▢ Your Dean of Students / Diversity Office/Director (DEI) (7)
- ▢ A Trusted faculty member/Career Counselor/ Academic Advisor (8)
- ▢ Other (Please Specify) (9)

---

Q3.3 How confident are you in your personal support structure to assist in your needs?

- ○ Not at all confident (1)
- ○ Unconfident (2)
- ○ Somewhat confident (3)
- ○ Confident (4)
- ○ Extremely Confident (5)

---

Q3.4 If you had a personal emergency, how confident are you in your school's ability to support you through your hardship?

- Not at all confident (1)
- Unconfident (2)
- Somewhat confident (3)
- Confident (4)
- Extremely Confident (5)

---

Q3.5 How much debt do you anticipate graduating medical school with?

- $400,000+ (1)
- $250,000 - $400,000 (2)
- $150,000 – $250,000 (3)
- $50,000 - $150,000 (4)
- (5)

---

Q3.6 If you are considering a VSLO (Visiting Student Learning Opportunity), how confident are you that you could pay for it?

- Not at all confident (1)
- Unconfident (2)
- Somewhat confident (3)
- Confident (4)
- Extremely Confident (5)

End of Block: Institutional Support

Start of Block: Demographic Questions

Section 5 Lastly, we would like to ask you a few questions about yourself.

Q5.1 Are you an Abigail Scholar? (Specific to GCSOM medical students)

- ○ No (1)
- ○ Yes (2)

Q5.2 What is the highest level of education attained by your parents or guardians?

- ○ Some high school or less (1)
- ○ High school diploma or GED (2)
- ○ Some college but no degree (3)
- ○ Associates or technical degree (4)
- ○ Bachelor’s degree (5)
- ○ Graduate or professional degree (MA, MS, MBA, PhD, JD, MD, DDS etc.) (6)

Q5.3 What was the average yearly income in your household while you were growing?

- Under $25,000  (1)
- $25,000 - $49,999  (2)
- $50,000 - $74,999  (3)
- $75,000 - $99,999  (4)
- $100,000 and above  (5)
- Prefer not to say  (6)

Q5.4 What year are you currently in medical school?

- M1 (First Year)  (1)
- M2 (Second Year)  (2)
- M3 (Third Year)  (3)
- M4 (Fourth Year)  (4)

Q5.5 How many dependents do you have?

- 0  (1)
- 1  (2)
- 2-3  (3)
- 4+  (4)

Q5.6 OPTIONAL: What will it mean to you when you can say 'I am a physician.'? (Open-ended) (1500 characters)

________________________________________________________________

________________________________________________________________

________________________________________________________________

________________________________________________________________

________________________________________________________________

**End of Block: Demographic Questions**
